# Anti-Phospholipase A2 Receptor (PLA2R) Antibody and Glomerular PLA2R Expression in Japanese Patients with Membranous Nephropathy

**DOI:** 10.1371/journal.pone.0158154

**Published:** 2016-06-29

**Authors:** Kei Hihara, Masayuki Iyoda, Shohei Tachibana, Ken Iseri, Tomohiro Saito, Yasutaka Yamamoto, Taihei Suzuki, Yukihiro Wada, Kei Matsumoto, Takanori Shibata

**Affiliations:** Division of Nephrology, Department of Medicine, Showa University School of Medicine, Tokyo, Japan; Postgraduate Medical Institute, INDIA

## Abstract

The phospholipase A2 receptor (PLA2R) is the major target antigen (Ag) in idiopathic membranous nephropathy (IMN). Recently, several types of immunoassay systems for anti-PLA2R antibody (Ab) have been developed. However, the correlation of serum anti-PLA2R Abs and glomerular expression of PLA2R Ag, and their association with clinicopathological characteristics have yet to be proven in Japanese patients. We examined serum anti-PLA2R Abs by both ELISA and cell-based indirect immunofluorescence assay (CIIFA), and glomerular PLA2R expression by immunofluorescence (IF) in 59 biopsy-proven MN patients including IMN (n = 38) and secondary MN (SMN) (n = 21). In this study, anti-PLA2R Abs were present in 50% of IMN patients, but was absent in SMN patients. The concordance rate between ELISA and CIIFA was 100%. Serum IgG levels were significantly lower in anti-PLA2R Ab-positive patients. Serum albumin levels correlated inversely with serum anti-PLA2R Ab titers. The prevalence and intensity of glomerular staining for IgG4 by IF were significantly higher in anti-PLA2R Ab-positive patients than in -negative patients. Glomerular PLA2 Ag expression evaluated by IF was positive in 52.6% of IMN patients, but was absent in SMN patients. The concordance rate between the prevalence of glomerular PLA2R Ag expression and anti-PLA2R Ab was 84.2%. The prevalence of anti-PLA2R Abs measured by ELISA/CIIFA was equivalent to previous Japanese studies evaluated using Western blotting. These analyses showed an excellent specificity for the diagnosis of IMN, and anti-PLA2R positivity was associated with some clinicopathological features, especially glomerular IgG4-dominant deposition.

## Introduction

Membranous nephropathy (MN) is the most common cause of nephrotic syndrome in the adult population. Approximately 75% of cases are idiopathic MN (IMN), and the remainder is secondary MN (SMN) induced by various clinical conditions including systemic lupus erythematosus (SLE), infections (hepatitis, syphilis), cancers, and drug exposure. IMN is an antibody-mediated autoimmune glomerular disease characterized by subepithelial immune deposits. Target antigens for IMN have been extensively explored to date, both experimentally and clinically. In Heymann nephritis, podocyte antigenic targets identified as megalin were shown to be responsible for *in situ* subepithelial immune complexes [1]. However, this antigen was not expressed in human podocytes. In 2002, Ronco’s group identified neutral endopeptidase (NEP) as the responsible antigen in a rare form of alloimmune antenatal MN [2].

In 2009, M-type phospholipase A2 receptor (PLA2R), a membrane glycoprotein localized to podocytes, was identified by Salant’s group as the target antigen in patients with IMN [3]. Studies have demonstrated that anti-PLA2R antibodies (Abs) ranged from 52 to 82% in patients with IMN [4], but were not detected in patients with secondary MN (SMN). During the clinical course of IMN, the degree of proteinuria and declining renal function were significantly associated with the serum anti-PLA2R antibody (Ab) level [5–10]. Thus, it has been suggested that serum levels of anti-PLA2R Ab are a potentially useful marker for the prediction of diagnosis and for monitoring the treatment response in IMN [5–10]. Anti-PLA2R Abs are primarily of the IgG4 subclass, which is consistent with the finding that most cases of IMN have IgG4-dominant subepithelial immune complexes. In Japan, the prevalence of anti-PLA2R Abs in IMN patients has been reported to be lower than in other countries [11,12].

The presence of anti-PLA2R Abs has been verified by various methods, including Western blotting, fluorescence immunoprecipitation assay (FIPA), ELISA, and cell-based indirect immunofluorescence assay (CIIFA). Recently, a commercially available ELISA based on purified human recombinant PLA2R and CIIFA utilizing fixed PLA2R-transfected HEK cells on slides as an antigenic substrate, were introduced by EUROIMMUN AG (Lubeck, Germany). Detection of anti-PLA2R Abs using ELISA was reported to have comparable sensitivity and specificity and correlate highly with the results of CIIFA in MN patients [13]. Although analysis for anti-PLA2R Abs is a useful tool for discrimination between IMN and SMN, taking serum samples at an appropriate time is important because the Abs can disappear after immunosuppressive therapy or spontaneous remission. On the other hand, histopathological analysis of glomerular PLA2R antigen (Ag) expression would be helpful to identify the PLA2R association at the time of renal biopsy when disease activity is high. Thus, we should consider PLA2R-related MN if patients have either anti-PLA2R Ab or enhanced glomerular PLA2R Ag expression [11].

In this study, we measured anti-PLA2R Abs using two different immunoassays (ELISA and CIIFA) and analyzed glomerular expression of PLA2R Ag in Japanese patients with MN whose sera were sampled at the time of renal biopsy before immunosuppressive treatment, and then correlated the prevalence with their clinicopathological characteristics.

## Materials and Methods

### Patients and samples

This study was approved by Ethics Committee of Showa University Hospital in Tokyo, Japan (No.1687) and patient data were anonymously used under consideration of the latest version of the Helsinki Declaration of human research ethics. All of the participants provided their written informed consent. We selected 38 patients with IMN and 21 patients with SMN who were admitted to Showa University Hospital between June 1993 and December 2014. All patients were diagnosed as MN according to the findings on renal biopsy. The diagnosis of IMN or SMN was based on renal biopsy findings and screening for underlying causes of SMN. Patients with IMN included 2 cases of relapse. Patients with SMN included 11 patients with systemic lupus erythematosus (SLE), 1 patient with hepatitis B virus (HBV) infection, 1 patient associated with bucillamine treatment, and 2 patients with cancer (breast cancer and gastric cancer). All of the renal biopsy procedures were performed before the initiation of immunosuppressive therapy. The two recurrent cases of IMN had not used any immunosuppressive therapy for at least 3 years. Blood samples and urine samples at the time of renal biopsy were collected from all of the patients. We also tested sera and urine in 12 patients with IMN sampled at least 1 year and 4 months after biopsy. Concerning triglyceride (TG), low-density lipoprotein cholesterol (LDL-chol) and high-density lipoprotein cholesterol (HDL-chol) measurement, we only compared cases where these parameters had been measured (TG: n = 58; LDL-chol: n = 49; HDL-chol: n = 49). The analysis of IgG subclasses in renal biopsy specimens was performed after 1996. Glomerular PLA2R expression in renal biopsy specimens were analyzed using stocked frozen or paraffin sections.

### Anti-PLA2R Ab measurement

Circulating anti-PLA2R Abs were measured in all patients using two different immunoassays, ELISA and CIIFA (EUROIMMUNE AG, Lübeck, Germany). Cut off values for ELISA and CIIFA were established according to the manufacture’s recommendations. The ELISA results were considered positive at a level > 20 U/ml for IgG anti-PLA2R Abs. The borderline was 14 to 20 U/ml. In this study, we have deemed the patients with borderline levels as being negative. In CIIFA, which uses transfected HEK 293 cells that express PLA2R and nontransfected cells as controls, negativity of anti-PLA2R Ab was defined as the absence of detectable Abs at a serum dilution of 1/10. Ab positivity was defined as positive staining at serum dilutions of 1/10 or higher.

### Renal biopsy

Renal biopsy specimens were divided and processed for light, immunofluorescence (IF) and electron microscopy (EM) analysis. For light microscopic study, tissues fixed in 10% aqueous formaldehyde solution (formalin) were embedded in paraffin using standard protocols. Paraffin-embedded materials were sectioned at 1 μm for routine staining with hematoxylin and eosin (H-E), periodic-acid Schiff (PAS) and Masson trichrome stains. Thin sections (0.5 μm thick) were used for periodic acid-methenamine silver stains (PAM). For IF, renal tissues were snap frozen in liquid nitrogen and cut into 3 μm sections. The glomerular deposits of IgG, IgA, IgM, C3, C4, C1q, fibrinogen, κ-light chain and λ-light chain in the biopsy specimens were examined.

In EM, we used the Ehrenreich and Churg classification for the ultrastructural staging of IMN.

### Glomerular PLA2 Ag expression

PLA2R expression was examined by indirect IF using rabbit polyclonal anti-PLA2R Abs (Sigma-Aldrich, St. Louis, MO, USA) at a dilution of 1:100, followed by Alexa Fluor 488-conjugated goat anti-rabbit IgG Abs (Invitrogen, Carlsbad, CA, USA) at a dilution of 1:100 [14].

### Glomerular IgG subclasses

Glomerular IgG subclass deposition was examined by direct IF using FITC-conjugated mouse anti-human IgG1, -IgG2, -IgG3, and -IgG4 monoclonal Abs as described earlier [15]. The fluorescence intensity of glomerular staining was graded as follows: negative, 0; very weak, 0.5; weak, 1; moderate, 2; strong, 3, and scored for each biopsy specimen.

### Statistical analysis

Data were recorded as means ± SD or medians (interquartile range) when appropriate. The *t* test, Mann-Whitney, and Fisher’s exact tests were used for comparison between groups. The correlation between several parameters was analyzed by Spearman’s rank coefficient of correlation. Cohen’s Kappa was used to analyze the agreement between the different immunoassays. Values of *P* < 0.05 were considered significant. All statistical analyses were performed using JMP version 11.0 for Windows.

## Results

### Baseline clinical characteristics

The clinical characteristics of the patients are shown in Table 1. Among the 59 patients with MN, 46% were male and 54% were female, with a median age of 61 years at the time of diagnosis. The mean serum albumin was 2.6 g/dl, and the mean proteinuria was 2.26 g/day. 37.3% of the patients had nephrotic syndrome (proteinuria ≥3.5 g/day and serum albumin ≤3.0 g/dl). Patients were divided into two groups: IMN (n = 38) and SMN (n = 21) (Table 1). There were no significant differences between IMN and SMN patients with respect to age, gender, serum creatinine, serum albumin, proteinuria, serum levels of IgA, IgM, TG, and LDL-chol, and the prevalence of nephritic syndrome. Serum IgG levels were significantly higher in the SMN patients and serum C3, C4, CH50, and HDL-chol levels were lower in the SMN patients compared to the IMN patients.

**Table 1:** Baseline clinical characteristics.

Characteristic	Total (n = 59)	IMN (n = 38)	SMN (n = 21)	P Value
Age (years)	61 (44–68)	64 (53–69)	44 (36–67)	0.17
Male gender	27 (45.8)	19 (50.0)	8 (38.1)	0.42
Serum creatinine (mg/dl)	0.70 (0.55–0.98)	0.74 (0.51–1.00)	0.68 (0.58–0.82)	0.63
Serum albumin (g/dl)	2.60 (2.20–3.40)	2.60 (2.20–3.33)	2.80 (1.85–3.50)	0.79
Proteinuria (g/day)	2.26 (0.85–3.60)	2.45 (0.91–4.38)	1.97 (0.67–3.05)	0.48
Serum IgG (mg/dl)	1025.0 (664.0–1427.0)	875.0 (562.8–1116.5)	1266.0 (943.0–1812.5)	<0.01
Serum IgA (mg/dl)	243.5 (196.0–317.5)	234.0 (193.5–308.5)	284.5 (196.8–330.8)	0.4
Serum IgM (mg/dl)	100.0 (55.8–151.8)	100.0 (63.0–156.8)	97.5 (51.8–148.3)	0.84
C3c (mg/dl)	123.0 (89.5–139.0)	129.2 (110.3–143.0)	83.4 (57.1–122.8)	<0.001
C4 (mg/dl)	28.1 (19.8–34.0)	30.8 (23.4–39.1)	15.2 (9.2–28.2)	<0.001
CH50 (U/ml)	43.8 (31.5–51.5)	45.2 (39.2–52.3)	31.0 (16.2–47.3)	<0.01
TG (mg/dl)	158.0 (116.0–231.0)	160.5 (117.8–270.3)	144 (95.0–205.0)	0.26
LDL-chol (mg/dl)	153.0 (111.5–221.0)	155.5 (118.5–221.0)	143.5 (87.8–232.3)	0.33
HDL-chol (mg/dl)	47.5 (41.8–74.3)	56.0 (44.5–79.5)	44.0 (39.0–59.0)	<0.05
Nephrotic syndrome	22 (37.3)	18 (47.4)	4 (19.1)	<0.05

Abbreviations: IMN, idiopathic membranous nephropathy; SMN, secondary membranous nephropathy; TG, triglyceride; LDL-chol, low-density lipoprotein cholesterol; HDL-chol, high-density lipoprotein cholesterol.

Values are given as the median (interquartile range) or number (%).

### Comparison of ELISA and CIIFA for the detection of anti-PLA2R Abs

We performed the anti-PLA2R Ab detection assay in 38 patients with IMN and 21 patients with SMN using commercially available ELISA and CIIFA diagnostic kits. Nineteen patients (50.0%) with IMN and no patients with SMN had positive anti-PLA2R Ab in each assay (Table 2). The concordance rate between the 2 methods was 100%.

**Table 2:** The prevalence of anti-PLA2R Abs evaluated by ELISA and CIIFA in MN patients.

		MN patients		
	anti-PLA2R Ab	IMN	SMN	Total
**ELISA**	**Positive**	19 (50%)	0 (0%)	19
	**Negative**	19 (50%)	21 (100%)	40
	**Total**	38	21	59
**CIIFA**	**Positive**	19 (50%)	0 (0%)	19
	**Negative**	19 (50%)	21 (100%)	40
	**Total**	38	21	59

Abbreviations: PLA2R, phospholipase A2 receptor; Ab, antibody; MN, membranous nephropathy; IMN, idiopathic membranous nephropathy; SMN, secondary membranous nephropathy; ELISA, enzyme-linked immunosorbent assay; CIIFA, cell-based indirect immunofluorescence assay.

Values are given as number (%).

### Clinical and histopathological differences between anti-PLA2R Ab-positive and -negative patients with IMN

**Fig 1:**
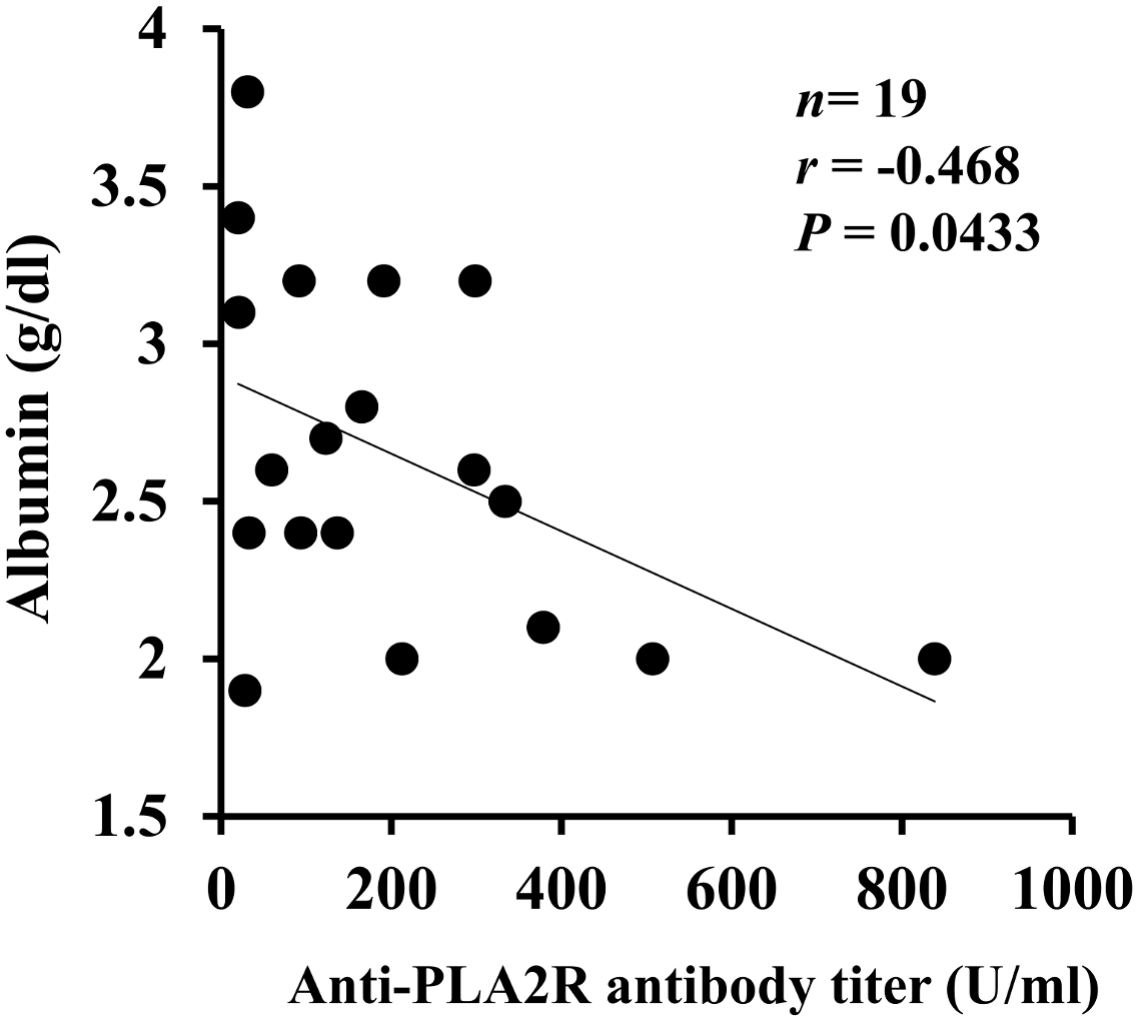
Correlation between serum albumin levels and serum anti-PLA2R Ab levels measured by ELISA.

We examined the clinical differences between serum anti-PLA2R Ab-positive and -negative patients with IMN (Table 3). There were no significant differences between the groups with respect to age, gender, serum creatinine, serum albumin, proteinuria, and the prevalence of nephrotic syndrome. Serum IgG levels were significantly lower in anti-PLA2R Ab-positive IMN patients than in -negative IMN patients (Table 3). Although we also analyzed the clinical parameters including C3, C4, CH50, TG, LDL-chol, and HDL-chol, no significant differences were detected between the study groups (data not shown). Serum albumin levels correlated inversely with serum anti-PLA2R Ab levels measured by ELISA (Fig 1). However, no correlation was detected between other clinical parameters and serum anti-PLA2R Ab levels (data not shown). Outcome data were available in 8 anti-PLA2R Ab-positive IMN patients and 4 anti-PLA2R Ab-negative IMN patients. Complete remission of proteinuria occurred in all of the 4 anti-PLA2R Ab-negative IMN patients and in 5 of 8 anti-PLA2R Ab-positive IMN patients. Two of 5 anti-PLA2R Ab-positive IMN patients who had complete remission were seronegative at the time of resolution of proteinuria.

**Table 3:** Clinical characteristics of the anti-PLA2R Ab-positive and -negative IMN patients.

	Anti-PLA2R Ab (n = 38)		
Characteristic	Positive (n = 19)	Negative (n = 19)	P Value
Age (years)	65 (58–73)	60 (41–67)	0.28
Male gender	9 (47.4)	10 (52.6)	>0.99
Serum creatinine (mg/dl)	0.70 (0.50–1.14)	0.80 (0.59–0.97)	0.91
Serum albumin (g/dl)	2.60 (2.10–3.20)	2.60 (2.20–4.10)	0.22
Proteinuria (g/day)	2.97 (1.60–4.50)	2.00 (0.47–4.36)	0.26
Serum IgG (mg/dl)	670.0 (520.0–992.0)	1063.0 (775.0–1469.0)	<0.05
Nephrotic syndrome	10 (55.0)	8 (42.1)	0.75

Abbreviations: PLA2R, phospholipase A2 receptor; Ab, antibody; IMN, idiopathic membranous nephropathy.

Values are given as median (interquartile range) or number (%).

Histopathologically, the extent of interstitial fibrosis and glomerular sclerosis was identical between the groups (Table 4). In IF, the prevalence of IgG4-positive staining in glomeruli and the intensity of IgG4 deposition were significantly higher in anti-PLA2R Ab-positive patients (Table 4). Among them, the prevalence of both IgG1- and IgG4-positive populations tended to be higher, whereas that of both IgG1- and IgG4-negative populations were significantly lower in anti-PLA2R Ab-positive patients. On the other hand, the prevalence or intensity of other IgG subclasses was identical between the groups. No significant differences were observed in EM stages between the groups (Table 4).

**Table 4:** Histopathological characteristics of the anti-PLA2R Ab-positive and -negative IMN patients.

	Anti-PLA2R Ab (n = 38)		
Characteristic	Positive (n = 19)	Negative (n = 19)	P Value
**LM**			
**Interstitial fibrosis**			
Mild	17/19 (89.4)	18/19 (94.7)	>0.99
Moderate	2/19 (10.5)	1/19 (5.3)	>0.99
Severe	0	0	0
**Glomerular sclerosis**			
Mild	7/19 (36.8)	9/18 (50.0)	0.515
Moderate	11/19 (57.9)	8/18 (44.4)	0.517
Severe	1/19 (5.3)	1/18 (5.6)	>0.99
**IF**			
IgG1 (+)	12/15(80.0)	8/13(61.5)	0.41
IgG2 (+)	6/15(40.0)	5/13(38.5)	>0.99
IgG3 (+)	4/15(26.7)	1/13(7.69)	0.33
IgG4 (+)	15/15 (100)	6/13 (46.1)	<0.01
IgG1 (+) / IgG4 (+)	12/15 (80.0)	5/13 (38.5)	0.051
IgG1 (-) / IgG4 (+)	3/15 (20.0)	1/13 (7.7)	0.6
IgG1 (+) / IgG4 (-)	0	3/13 (23.1)	0.09
IgG1 (-) / IgG4 (-)	0	4/13 (30.8)	<0.05
IgG1 intensity	1.40 ± 0.91	1.15 ± 1.06	0.53
IgG2 intensity	0.47 ± 0.64	0.54 ± 0.78	0.92
IgG3 intensity	0.27 ± 0.46	0.08 ± 0.28	0.2
IgG4 intensity	2.53 ± 0.92	1.08 ± 1.26	<0.01
**EM**			
I and I-II	2/17 (11.8)	6/17(35.3)	0.22
II and II-III	12/17 (70.6)	7/17(41.2)	0.17
III,III-IV and IV	3/17 (17.7)	4/17(23.5)	>0.99

Abbreviations: PLA2R, phospholipase A2 receptor; Ab, antibody; IMN, idiopathic membranous nephropathy; LM, light microscopy; IF, immunofluorescence; EM, electron microscopy.

Values are given as mean ± SD or number (%).

### Glomerular PLA2R Ag expression

We analyzed the glomerular expression of PLA2R Ag in 38 patients with IMN and 17 patients with SMN. Twenty patients (52.63%) with IMN and no patients with SMN had positive glomerular PLA2R Ag expression (Table 5). There were no significant differences between glomerular PLA2R Ag expression-positive and -negative IMN patients with respect to age, gender, serum creatinine, serum albumin, proteinuria, and the prevalence of nephrotic syndrome (data not shown). Serum IgG levels tended to be lower in glomerular PLA2R Ag expression-positive IMN patients than in -negative IMN patients, however this trend did not reach statistical significance (733.0 [520.8–1017.8] vs 1063.0 [670.0–1359.0] mg/dl, *P* = 0.08). Histopathologically, the prevalence and the intensity of IgG4 were significantly higher in glomerular PLA2R Ag expression-positive patients than -negative patients (prevalence: 100 vs 41.7%, *P*<0.01; intensity: 2.60±0.91 vs 0.92±1.16, *P*<0.01). In agreement with the results of anti-PLA2R Abs, no significant differences were observed in the extent of interstitial fibrosis and glomerular sclerosis, and EM stages between the groups (data not shown).

**Table 5:** The prevalence of glomerular PLA2R Ag expression in MN patients.

	MN patients		
	IMN	SMN	Total
**Glomerular PLA2R Ag expression**			
**Positive**	20 (52.63%)	0 (0%)	20
**Negative**	18 (47.37%)	17 (100%)	35
**Total**	38	17	55

Abbreviations: PLA2R, phospholipase A2 receptor; Ag, antigen; MN, membranous nephropathy; IMN, idiopathic membranous nephropathy; SMN, secondary membranous nephropathy.

Values are given as number (%).

### Comparison of the prevalence of serum anti-PLA2R Abs with glomerular PLA2R Ag expression

We compared the prevalence of serum anti-PLA2R Abs detected by ELISA/CIIFA with glomerular PLA2R Ag expression in IMN patients (Table 6). Seventeen of 20 IMN patients with positive glomerular PLA2R expression were positive for anti-PLA2R Abs. On the other hand, 2 in 18 IMN patients with negative glomerular PLA2R Ag expression were positive for anti-PLA2R Ab. The overall qualitative agreement was 84.2% (95% CI: 67.8–100.6%).

**Table 6:** Comparison of the prevalence of serum anti-PLA2R Abs with glomerular PLA2R Ag expression in IMN patients.

	Glomerular PLA2R Ag expression (n = 38)		
anti-PLA2R Ab	Positive (n = 20)	Negative (n = 18)	Total
**Positive (n = 19)**	17 (44.74%)	2 (5.26%)	19 (50.0%)
**Negative (n = 19)**	3 (7.89%)	16 (42.11%)	19 (50.0%)
**Total**	20 (52.63%)	18 (47.37%)	38
**Kappa (95% CI)**	0.737 (0.52–0.95)		

Abbreviations: PLA2R, phospholipase A2 receptor; Ab, antibody; Ag, antigen; IMN, idiopathic membranous nephropathy; 95% CI, 95% confidence interval.

Values are given as number (%).

## Discussion

In this study, the prevalence of anti-PLA2R Abs evaluated by ELISA/CIIFA was 50% in IMN patients before the initiation of immunosuppressive therapy, which was lower compared to studies in other countries ranging from 52% to 82% [4]. The concordance between CIIFA and ELISA performed in the present study was 100%. The reason for the low prevalence of anti-PLA2R Abs in Japanese IMN patients has been speculated to be due to genetic differences, diagnosis at an early stage of the disease due to the efficient health check-up system, environmental or dietary factors, involvement of other pathogenic antigens, and differences in immunoassays [12]. In fact, the prevalence of nephrotic syndrome and the average level of proteinuria in this study were 47.4% and 2.45 g/day, which were much lower than previous studies from foreign countries that ranged from 75 to 93% and from 6.1 to 10.2 g/day, respectively [5,7,16]. Although anti-PLA2R Ab titers were not statistically correlated with proteinuria, which could be due to low average level of proteinuria and/or small sample size, the titers were weakly inversely correlated with serum albumin levels. It is possible some auto-Abs are more pathogenic so could cause more severe nephrotic syndrome/hypoalbuminemia even at low titers. There are several reports that have demonstrated the close association of anti-PLA2R Abs with clinical status [5,17,18]. The group of Salant and co-workers showed anti-PLA2R Ab levels were high in the initial phase of nephrotic syndrome, decreased significantly during remission, and increased again during relapse [5]. In the IMN patients who received rituximab, an anti-CD20 monoclonal Ab, the decline in anti-PLA2R Ab preceded the corresponding changes in proteinuria [17,19]. In our study, the number and percentage of IMN patients who are anti-PLA2R positive in nephrotic patients vs. non-nephrotic patients were 10/18 (55.6%) vs. 9/20 (45%). Accordingly, we believe the selection of a population with significantly different proteinuria is likely contributing to the different prevalence of anti-PLA2R Abs in IMN patients.

On the other hand, our results were identical to other studies in Japan despite the use of a different immunoassay; namely, 53% reported by Akiyama et al. [12] and 55% reported by Hayashi et al. [11], both of which were evaluated using Western blotting. Thus, we consider the clinical utility of ELISA, CIIFA, and Western blotting for the detection of anti-PLA2R Abs to be equivalent. Of note, none of the SMN patients had anti-PLA2R Abs positivity, thus the analysis for anti-PLA2R Abs are highly useful in the discrimination between IMN and SMN. Anti-PLA2R related IMN is much more common in males. Unlike the 2 other Japanese studies [11,12], in this study of Japanese patients, males and females were equivalent. Perhaps this could be due to small sample size.

Other target antigens, such as aldose reductase (AR), superoxide dismutase-2 (SOD2), and, α-enolase (αENO) [20–22] are potentially involved in the development of anti-PLA2R Ab-negative IMN patients. Murtas et al. reported that IgG4 Abs against AR, SOD2, αENO were detected in 34%, 28%, 43% of patients, respectively, and correlated with anti-PLA2R IgG4 Abs in IMN patients at diagnosis [23]. Approximately half of the anti-PLA2R Ab-negative IMN patients were also negative for other Abs, and these populations were associated with lower proteinuria 1 year later [23]. Thrombospondin type-1 domain-containing 7A (THSD7A) is a newly identified target antigen, with Abs to THSD7A being predominantly of the IgG4 subclass [24]. About 8 to 14% of anti-PLA2R Ab-negative IMN patients had anti-THSD7A Abs, whereas none of the serum samples from patients with seropositive for anti-PLA2R Abs reacted against THSD7A [24]. Recently, Iwakura et al. reported the higher prevalence of enhanced glomerular THSD7A Ag expression in Japan compared to USA or Europe [25], which could be a possible explanation in part of the lower prevalence of anti-PLA2R Abs in the Japanese population.

On the other hand, Beck and Salant speculated that the anti-PLA2R Ab negativity was a result of an absence of immunologic disease activity at the time of serum sampling [26]. A recent report supports the notion that anti-PLA2R Ab- and anti-THSD7A Ab-negative patients with IMN have a high rate of spontaneous remission of proteinuria [27]. In this study, proteinuria was relatively high and serum IgG levels were significantly lower in anti-PLA2R Ab-positive IMN patients, which is consistent with a previous report [11] and might represent the low clinical activity of the anti-PLA2R Ab seronegative population. In addition, complete remission of proteinuria occurred in all of the 4 anti-PLA2R Ab-negative IMN patients and in 5 of 8 anti-PLA2R Ab-positive IMN patients during follow-up. Two of 5 anti-PLA2R Ab-positive IMN patients who had complete remission were seronegative at the time of resolution of proteinuria. These data indicate that anti-PLA2R Ab seropositive patients are clinically active.

Another possibility is that anti-PLA2R Ab negativity reflects the early stage of IMN. Huang et al. indicated the possibility of IgG class switch from IgG1 to IgG4 in accordance with the progress of the disease, based on the observation that IgG1 was the dominant IgG subclass in stage I and IgG4 dominated in later stages II-IV [28]. This can explain the differences in certain clinicopathological features between the anti-PLA2R Ab-positive and -negative IMN patients in this study. First, the prevalence and the intensity of IgG4 staining in glomeruli were significantly higher in anti-PLA2R Ab-positive patients than in -negative patients. Of note, IgG4 positivity in anti-PLA2R Ab-positive patients was 100%, whereas it was 46.1% in -negative patients. Second, IgG1 positivity without IgG4 was observed only in anti-PLA2R Ab-negative IMN patients. Third, though the number of patients is small, anti-PLA2R Ab-negative patients were highly prevalent in stage I and I-II (35.3% of cases) compared to -positive patients (11.8% of cases). However, these evidences are not enough to say that PLA2R-related MN first manifests as anti-PLA2R Ab-negative and IgG1 dominant. It is possible that the difference in IgG4 positivity just reflects the fact that IMN could be separated into two distinct groups: PLA2R-related MN and non-PLA2R-related MN. In fact, Kattah et al. reported that all of 5 post-transplant recurrent anti-PLA2R Ab-positive IMN patients had predominantly IgG4 staining [29].

Glomerular PLA2R Ag expression evaluated by IF with the use of polyclonal rabbit PLA2R Ab was positive in 52.63% (20/38) of IMN patients, but was absent (0/17) in SMN patients. Previous studies reported the prevalence of enhanced glomerular PLA2R Ag staining was 63.6% (14/22) in the Japanese population [11], 69.3% (61/88) [30], 73.8% (31/42) [31], and 69.2% (45/65) [32] in European populations, and 52.2% (36/69) in the Chinese population [33]. The concordance rate between glomerular PLA2R and the prevalence of anti-PLA2R Ab measured by ELISA/CIIFA in this study was 84.2%. This high concordance rate could be attributed to the fact that sera were sampled at the time of renal biopsy before immunosuppressive treatment. Two IMN patients with positive-anti-PLA2R Ab was negative for glomerular PLA2R Ag expression. One IMN patient was a case of IMN relapse after 16 years. The anti-PLA2R Ab level was relatively high at 136 U/ml, urinary protein was 6.55 g/day, and EM stage was III-IV. We speculated that this was a case of very early stage relapse in consideration of the EM findings, which had yet to show any supplementary subepithelial deposits. Another explanation is that tissue can be false negative for PLA2R in late stage IMN because deposits are clearing out and lose antigenicity, but the patient can have persistent heavy proteinuria. The other patient was an initial case of IMN with 3.20 g/day of proteinuria, 334 U/ml of anti-PLA2R Ab, and an EM stage of I, indicating a case of very early stage. The patient’s proteinuria decreased to 0.23 g/day and the PLA2R Ab turned to be negative with prednisolone treatment. On the other hand, 3 IMN patients with negative-anti-PLA2R Ab were positive for glomerular PLA2R Ag expression. One IMN patient was a case of IMN relapse after 40 years with 0.35 g/day of proteinuria and an EM stage was II and IV. The other patients were initial cases of IMN with 2.64 and 3.60 g/day of proteinuria and an EM stage of I-II and II, respectively. Debiec et al. speculated that these discordant findings might be due to the rapid clearance of Abs or to the late serum sampling when Abs have already disappeared, even though proteinuria persisted, which was due to irreversible ultrastructural changes [31]. There may be a time discrepancy between the appearance of anti-PLA2R Ab and glomerular PLA2R Ag; therefore, the absence of anti-PLA2R Ab dose not rule out a diagnosis of PLA2R-related MN [31,32]. It seems that anti-PLA2R Ab precedes glomerular PLA2R Ag expression and the glomerular expression occasionally persists even if the Ab disappears. We should consider PLA2R-related MN if patients have either anti-PLA2R Ab or enhanced glomerular PLA2R Ag expression [11]. Finally, we found 22 PLA2R-related MN patients (57.89%) in this study that was still lower than the prevalence of anti-PLA2R Ab positive IMN patients in other countries [4].

In conclusion, the prevalence of anti-PLA2R Abs and glomerular PLA2R Ag expression was confirmed in a Japanese IMN population, and these analyses showed an excellent specificity for the diagnosis of IMN. The prevalence of anti-PLA2R Abs evaluated using commercially available ELISA/CIIFA kits was 50% with excellent agreement between both immunoassays, which was equivalent to a previously reported Japanese study evaluated using Western blotting. These analyses were associated with some clinicopathological features, especially glomerular IgG4-dominant deposition.
